# Design and Implementation of Intelligent Sports Training System for College Students' Mental Health Education

**DOI:** 10.3389/fpsyg.2021.634978

**Published:** 2021-04-08

**Authors:** Ting Wang, Jinkyung Park

**Affiliations:** College of Tourism and Sports, Catholic Kwandong University, Gangneung-si, South Korea

**Keywords:** intelligent sports system, college students' psychological education, deep learning, recommendation algorithm, artificial intelligence

## Abstract

In order to solve the problems of poor physical fitness of college students and low efficiency of college sport venues' management, an intelligent sports management system based on deep learning technology is designed by using information technology and human-computer interaction under artificial intelligence. Based on the Browser/Server (B/S) structure, the intelligent sports management system is constructed. The basic framework of Spring Cloud is used to integrate the framework and components of each part, and a distributed microservice system is built. The artificial intelligence recommendation algorithm is used to analyze the user's age, body mass index (BMI), and physical health status, and recommend sports programs suitable for students, thus realizing the intelligent sports program recommendation function. At the same time, the recommendation algorithm is used to complete the course recommendation according to the students' preferences, teaching distance, opening time, course evaluation, and other indexes, and the course registration system is constructed; after the analysis of the entity and the relationship between the entities of the intelligent sports system, the database relational model of the system is designed with the entity relationship (E-R) diagram. The results of the functional test show that the system can run well. In conclusion, the sports training environment instructional system based on artificial intelligence and deep learning technology can meet the teaching needs of colleges, improve the sports' quality for college students, and promote psychological education.

## Introduction

On October 12, 2019, the Ministry of Education issued the *Opinions on Deepening Undergraduate Education and Teaching Reform and Comprehensively Improving the Quality of Personnel Training*. This opinion requires that the examination and graduation exit of colleges should be strictly controlled, the assessment of students' physical education courses should be strengthened, and those who fail to meet the requirements could not graduate. The results of the seventh national survey on students' physique and health have been published. The data show that the physical fitness of college students continues to show a downward trend. In particular, the speed, explosive power, endurance, and other physical fitness indexes of male students aged 19–22 years old have decreased (Chen, 2019). Other studies show that a lack of sleep is common among college students. As high as 43.9% college students sleep fewer than 7 h a day, and 8.4% of them sleep <6 h; moreover, the sleep habits of college students are also worrying. 23.8% college students fall asleep after midnight, and the average bedtime is close to 1:00 a.m. (Qian et al., 2018). Mental health problems are common amongst college students. College students' anxiety is more common in China, and the detection rate is on the rise.

Strengthening exercises are key to improving the physical and mental health of college students (Wu et al., 2019). College is the transition stage of the mind from immaturity to maturity, and the cultivation of a healthy mental state in college students has attracted much attention (Wu and Wu, 2017). The study shows that, compared with college students who actively participate in sports, the risk of obesity among college students who lack exercise is 1.25 times higher (Zheng et al., 2018). Sports are essential to improve the mental health of college students, including sleep quality, anxiety, and depression of college students, and this has been confirmed by many evidence-based medical studies (Zhao and Yang, 2019). College campuses have unique sports resources but fail to prevent the lack of sports activities and mental problems of college students. Sports venues are an indispensable part of the teaching infrastructure of colleges, and their most basic function is to protect the physical and mental health of students (Li and Qu, 2019). The problems with sports teaching in colleges have existed for a log time. In recent years, due to the annual expansion of college enrollment, the number of teachers and amount of sports equipment is facing a shortage problem (Wu and Song, 2019). Many local colleges and universities lack enough diverse sports venues, and the inefficient sports venues and training environment system makes more students unwilling to carry out sports training and exercise (Wu et al., 2020). Using artificial intelligence technology to meet the development needs of education modernization and promote the transformation and reconstruction of education mode has become a widespread practice in the field of education.

Therefore, based on artificial intelligence information technology and deep learning technology, an intelligent sports management system is designed. The key technologies are introduced and analyzed, the system requirements are analyzed, and the database of intelligent sports management system is established. Based on the actual application needs, some problems and inconveniences existing in the management of sports venues and college students' sports training and teaching environment are solved, the required human and material resources are reduced, and the operation efficiency of sports venues are improved, so as to provide convenient and intelligent services for college students, improve the frequency of sports training and exercise of college students, and promote physical and mental health development.

## Method

### The Architecture of a Sports Management System Based on Artificial Intelligence and Human-Computer Interaction

Deep learning has become a research hotspot in the field of artificial intelligence. Various research results based on deep learning have been applied in practice. It is a method of learning data representation through machine learning. The recognizable feature representation can be obtained through learning multi-level combination, and finally mapped to the task target. With the idea of end-to-end interaction, a deep learning model can directly convert input into output. The process of feature extraction and feature mapping to target output are automatically completed by the model, which saves on the many tedious intermediate processes in traditional machine learning. Artificial intelligence is developing rapidly in various fields. The field of efficient sports is very suitable for the development of artificial intelligence because of its wide audience and the need for professional guidance.

For the architecture of an intelligent sports management system, there are a variety of technologies and methods to choose from. Native is a native application development mode, which can make full use of the functions of the software platform and API interface. The main advantages of Native are smooth running, high stability, and low energy consumption. However, there are some problems in cross platform development. Programmers and staff need to spend time and energy to solve the portability problem in cross platform development. It will take some time to solve this problem, and there are certain restrictions on the development, so the browser-based development mode is selected in this system.

Intelligent wearable devices are another innovative form of modern intelligent technology, and can be used to directly obtain a comprehensive index of the human body. In sports teaching, wearable devices based on human-computer interaction can be used to measure the body indexes of students in different sports states. Moreover, the use of intelligent wearable devices can enhance students' interest in sports and change their cognition of the limitations of sports (Yang et al., 2020). Based on the application of human-computer interaction equipment, the construction of sports management systems needs to combine artificial intelligence and deep learning technology.

**Figure 1:**
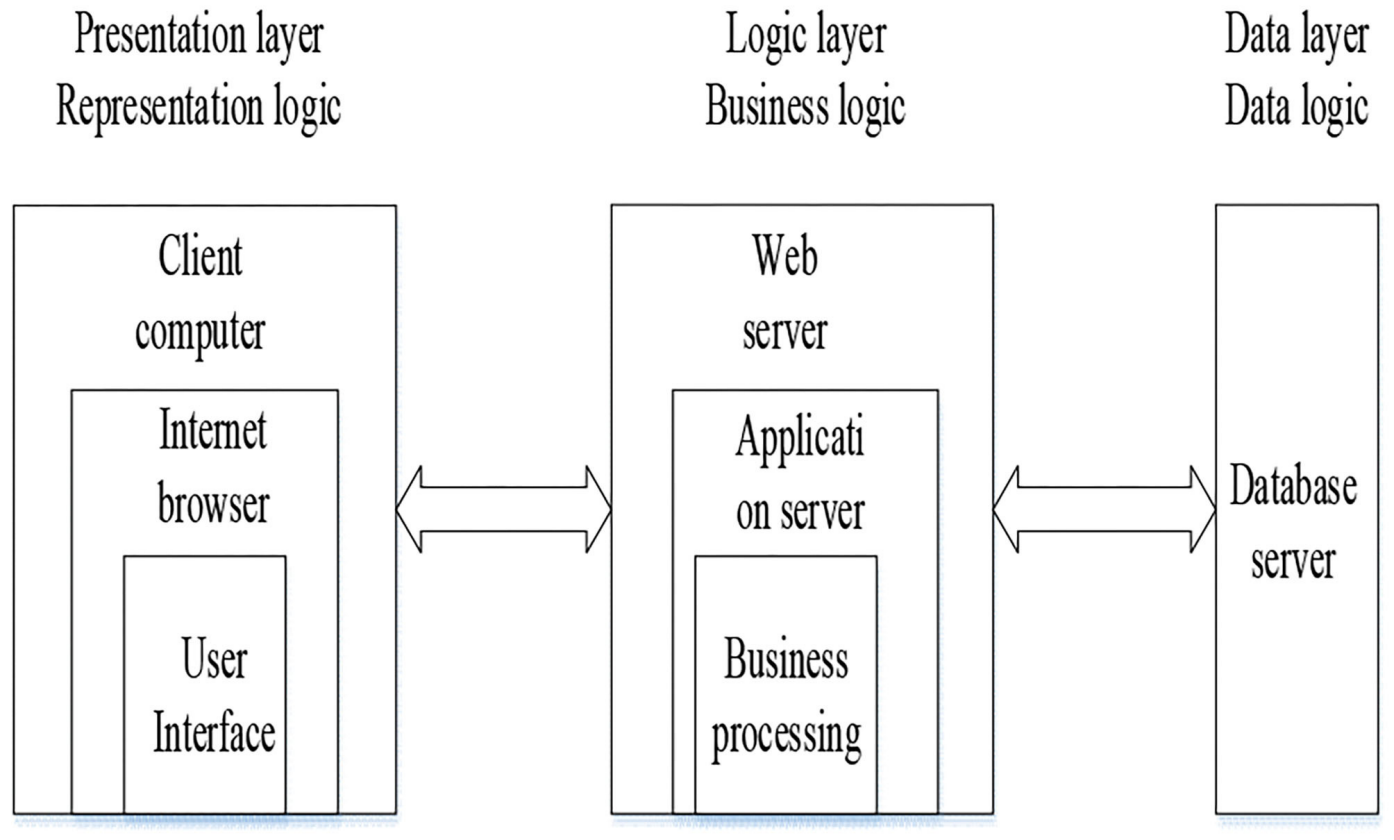
B/S structure diagram.

Artificial intelligence education environment system is based on a new generation of information technology, such as deep learning algorithm, to build a learner-centered intelligent education environment throughout all aspects of the education process, and to achieve a more diverse, more accurate, and more personalized education environment (Zhao et al., 2019). In an intelligent sports management system, the Browser/Server (B/S) structure is adopted, which divides the system application into presentation layer, business logic layer, and data access layer (Yan et al., 2019). Among them, according to the top-down level in the three layers, the presentation layer is used to display the data and receive the data transmitted from the user from the outside to the internal, which is the interface closest to the user. Then, the business logic layer operates the data layer, which plays an important role in the whole system. The bottom layer is the data access layer, which operates the database (Kravari and Bassiliades, 2019). The working principle of B/S structure is to connect to the database Server in many ways. Users send their own access requests to the Web server through the Browser. The Web server accepts and processes the requests, and then responds to the Browser. Finally, the Browser receives the response, parses and arranges relevant resource files, and displays the user page (Yi et al., 2019). B/S structure has many advantages, and any user scale will not affect the workload of maintenance and upgrading (Li et al., 2019). Therefore, the B/S three-tier structure is selected as the software architecture of the system, which simplifies the development, maintenance, and use of the system (Gómez et al., 2020). Figure 1 shows the three-tier structure of B/S.

### Artificial Intelligence Microservice Architecture Based on Deep Learning and Spring Cloud Method

In artificial intelligence technology, microservice architecture is used to decompose the original huge services into multiple microservices. Among them, each microservice can run independently and communicate with HTTP-based API using lightweight devices (Yin et al., 2020). Microservices and distributed architecture are becoming more and more popular, and Spring Boot based on REST is just such a service framework. The core of Spring Boot framework is automatic configuration. Using common frameworks in zero XML configuration greatly simplifies the development mode, improves the development efficiency, and shortens the development cycle of the project (Lin et al., 2019). Therefore, in this system, Spring microservice architecture is adopted to decompose the huge service into multiple microservices.

**Figure 2:**
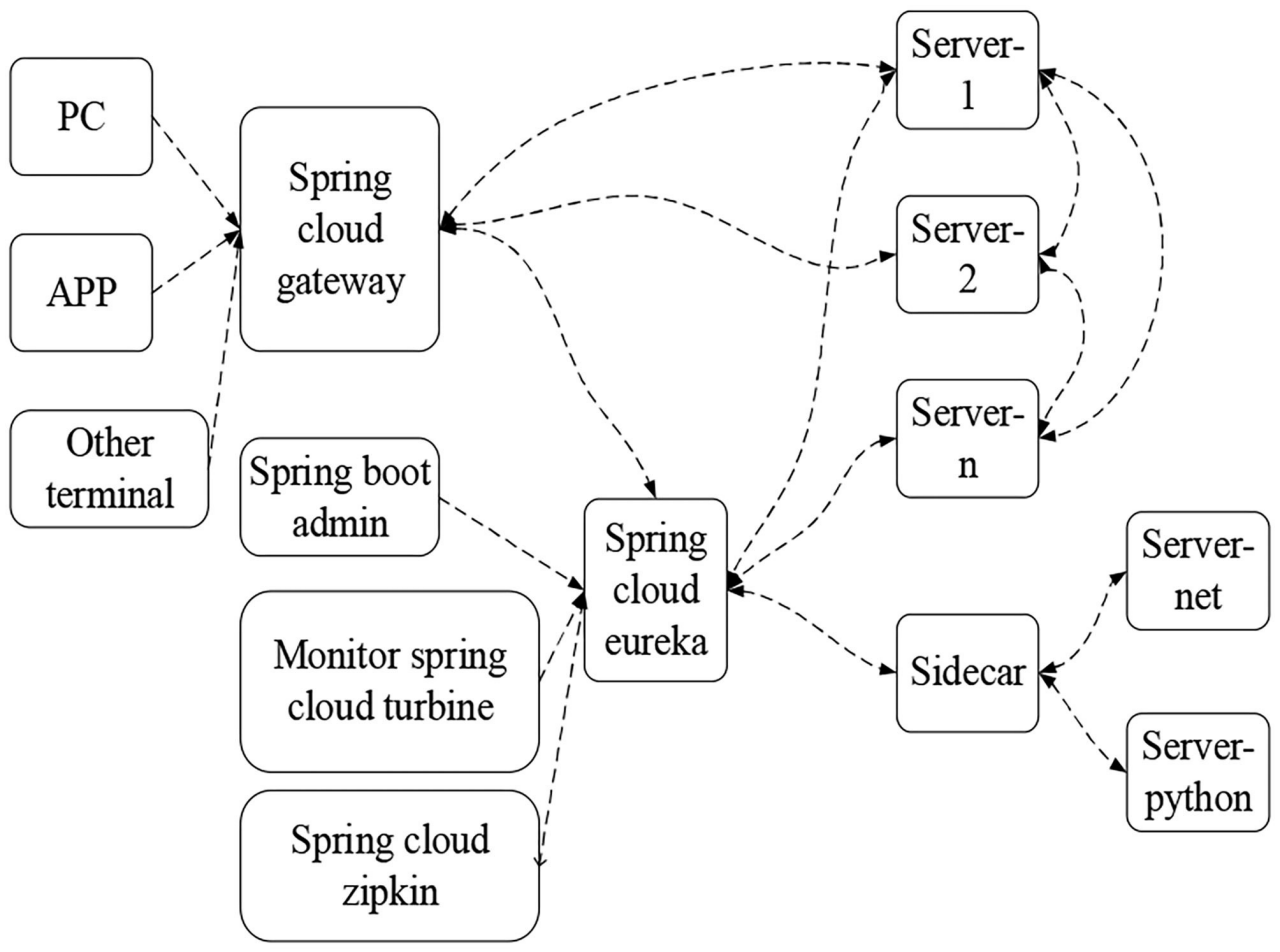
Spring Cloud component architecture.

The development of deep learning technology is to promote the development of artificial intelligence. Therefore, there is a cloud server suitable for deep learning. In a deep learning network, each node layer learns to recognize a set of specific features based on the output of the previous layer. As the depth of the neural network increases, the features that nodes can recognize become more and more complex, because each layer will integrate and reorganize the features of the previous layer. Spring Cloud architecture based on deep learning technology can provide services perceptively. Spring Cloud is the epitome of microservice architecture based on the Spring Boot framework, and provides strong backing for microservice architecture. It is an aggregation of all components (Tian et al., 2019). To reduce the difficulty for users to build and maintain distributed systems, Spring Cloud supports a set of development tools for many common functions and cluster state management (Leffler et al., 2019; Morrison et al., 2019; Vaughn et al., 2019). Therefore, in this system, Spring Cloud is selected to let developers quickly build distributed microservice systems through the integration of good framework and components. The advantages of Spring Cloud are as follows: the maintainability of the system can be effectively improved through the improvement of the whole service project planning; the use threshold is relatively low; and due to the fast update speed of products, they are more popular in today's Internet era. Figure 2 shows the component architecture of Spring Cloud.

Redis is a high performance Key-Value distributed memory database. Its source code is open source. It can support persistent logs and store a variety of data structures of different value types. This system uses Redis to cache data. The advantages of Redis mainly include the following points. Its operation object is the data in the memory, so its reading and writing speed is quite fast and the performance is very high. It supports multiple data types. The advantage of Redis is not only its performance, but also its ability to support multiple data structures, which is more attractive. In addition, compared with memcached, it has more than 1,000 times the maximum data storage capacity, so it can achieve more functions. It also supports a variety of persistence solutions to solve the problem of data loss in the event of memory database failure. In view of the above advantages, Redis technology is most suitable for caching. It reduces the read operation to the database to reduce the pressure of the server, and improves the response speed of the system.

Maven is a software tool for building and managing Java related projects. In the development process of a software project, developers need to carry out the same repeated steps: code editing, unit testing, packaging, publishing the project, and writing documents. These repetitive tasks create a high workload for developers. The emergence of Maven greatly facilitates the work of developers, who can focus on business logic and implementation. Therefore, in this system, Maven is used for project management.

### Artificial Intelligence Recommendation Algorithm

In an intelligent sports management system, based on the construction of sports health knowledge ontology database, a hybrid intelligent sports scheme recommendation model is designed, and the similarity calculation algorithm used in solving the model is introduced.

Content-based recommendation is based on whether users liked the items in the past, to make a detailed analysis and obtain the characteristic attributes of the items that users like. Finally, it recommends similar items to users based on this feature attribute (Agans et al., 2020). Fitness equipment is used as an example. First, it is necessary to extract the attribute features from the content of fitness equipment. Which body parts can be exercised by the equipment is taken as feature attribute of the equipment. TF-IDF model is used to calculate the weight of each part (Li and Ning, 2020).

The vector model that defines the content of an item is as follows.

(1) $$\begin{array}{r} Content\left(i\right)=\left\{Q_{i1},Q_{i2},...\right\} \end{array}$$

Among them, *Q*_*ik*_ represents the weight of item *i* in the *K* position.

The vector expression that defines user *m* is as follows:

(2) $$\begin{array}{r} Profile\left(m\right)=\frac{1}{\left|S\left(m\right)\right|}\sum_{i\in S\left(m\right)}Content\left(i\right) \end{array}$$

Among them, *S*(*m*) represents the item category selected by the user in the past.

Recommendations based on collaborative filtering first analyze users' interests and hobbies, find users with similar interests and hobbies, and then predict the interests of target users according to the preferences of similar users for some items, and finally recommend the items with a high prediction score to the target users (Renó et al., 2019).

In terms of similarity calculation equation, cosine similarity, co-occurrence similarity, and Pearson correlation coefficient are briefly introduced and used.

The calculation method of cosine similarity is to calculate the cosine value of the angle between two vectors, and take the cosine value as the basis to consider the similarity. Here, two n-dimensional vectors are shown as examples. If there are two vectors $\vec{a},\vec{b}$, the cosine similarity between $\vec{a}$ and $\vec{b}$ can be expressed as follows.

(3) $$\begin{array}{r} sim\left(a,b\right)=0.5+0.5\frac{\vec{a}*\vec{b}}{\left|\vec{a}|*|\vec{b}\right|} \end{array}$$

The calculation equation of co-occurrence similarity is as follows.

(4) $$\begin{array}{r} W_{a.b}=\frac{\left|N\left(a\right)\cap N\left(b\right)\right|}{\sqrt{\left|N\left(a\right)||N\left(b\right)\right|}} \end{array}$$

N(a) is the number of users who select item *a*, and |*N*(*a*)∩*N*(*b*)| is the number of users who select both *a* and *b*.

Pearson correlation coefficient is used to calculate the degree of closeness between the two variables. The equation is as follows.

(5) $$\begin{array}{r} p\left(x,y\right)=\frac{\mathrm{cov}\left(X,Y\right)}{\sigma_{X}\sigma_{Y}}=\frac{\overset{n}{\underset{i=1}{\sum }}\left(X_{i}-\bar{X}\right)\left(Y_{i}-\bar{Y}\right)}{\sqrt{\overset{n}{\underset{i=1}{\sum }}{\left(X_{i}-\bar{X}\right)}^{2}}\sqrt{\overset{n}{\underset{i=1}{\sum }}{\left(Y_{i}-\bar{Y}\right)}^{2}}} \end{array}$$

Its value range is between [−1,1]. When the value is 1, it indicates that the two variables are positively correlated, which also means that the similarity is very high; when the value is 0, it indicates that the two variables have no relationship, which also means that the similarity is very low.

### Construction of Artificial Intelligence Sports Scheme Recommendation Model

In order to realize the function of sports scheme making and recommendation in an intelligent sports management system, the specific field of sports health is selected for detailed understanding, research, and modeling. Combined with the information of users, such as age, height, weight, and health status, the sports scheme is generated and recommended to users.

**Figure 3:**
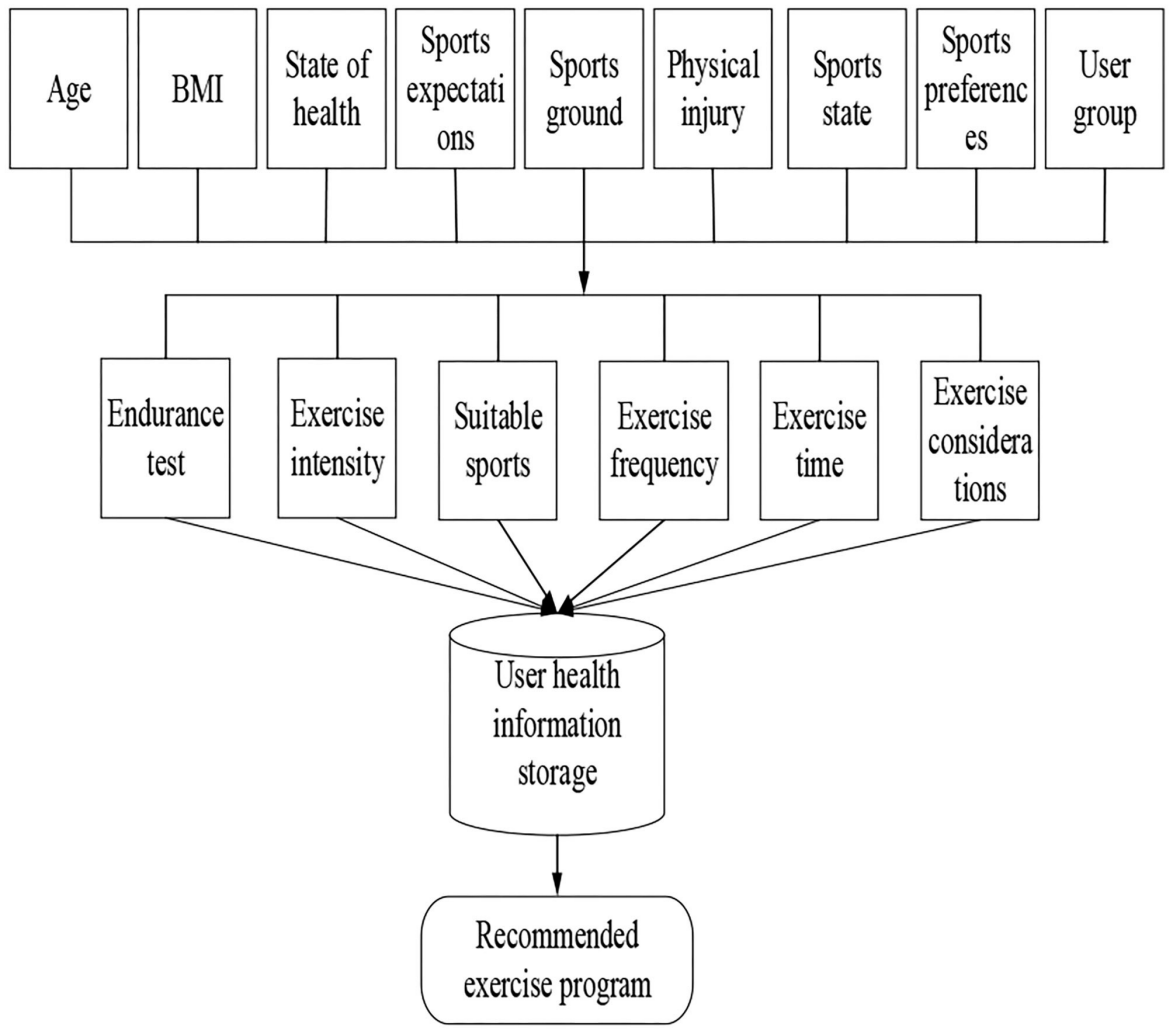
Sports scheme recommendation model.

The age, BMI index, and health status of users are analyzed, and the same sports scheme is recommended to users. Based on the traditional cooperative recommendation algorithm, combined with the knowledge of sports health, a case reasoning model based on ontology similarity calculation is designed to realize the recommendation function of sports scheme in intelligent sports management system. By analyzing the mature sports scheme cases in sports rehabilitation and sports prescription, and combining with the advice of doctors and health care professionals, the recommendation model is designed. Figure 3 is the sports scheme recommendation model.

### Demand Analysis of Artificial Intelligence Sports Training Environment System

At present, most college sports venues are mainly operated and managed manually. Personnel registration and reservation work lead to increased costs and high error rates (Bharathi and Selvarani, 2019). For sports that both teachers and students like, the venue is usually full, so if students want to exercise on the field, they must make an appointment as soon as possible. However, the venue management personnel and users cannot accurately grasp the real-time information of the site status. As a result, some idle venues have no one to exercise, some venues are full of staff, and resources cannot be reasonably utilized, which cannot ensure the efficient operation of venues. Therefore, it is urgent for researchers to develop an advanced information management software system for college sports training.

The system requirements mainly include the following points.

(1) It must have basic functions, such as user registration and login.
(2) It supports the user to book in the browser.
(3) It can add, modify, and delete user information in the background management.
(4) It can give different discount functions according to different roles.
(5) It can let users see the status of all the site information at a glance, and the administrator can operate the site on and off the shelf.
(6) It allows users to reserve parking spaces and venues.
(7) It allows users to rent and return fitness equipment.
(8) It allows the administrator to manage information and generate reports.
(9)It recommends fitness equipment and sports schemes to users according to the combination recommendation algorithm.

### Database Structure of Artificial Intelligence System

Database design is an important part of system requirement analysis and design. It is directly related to whether the system can be realized and the performance after implementation. In software engineering, database design determines the data storage structure in software development according to the needs of the first party.

**Figure 4:**
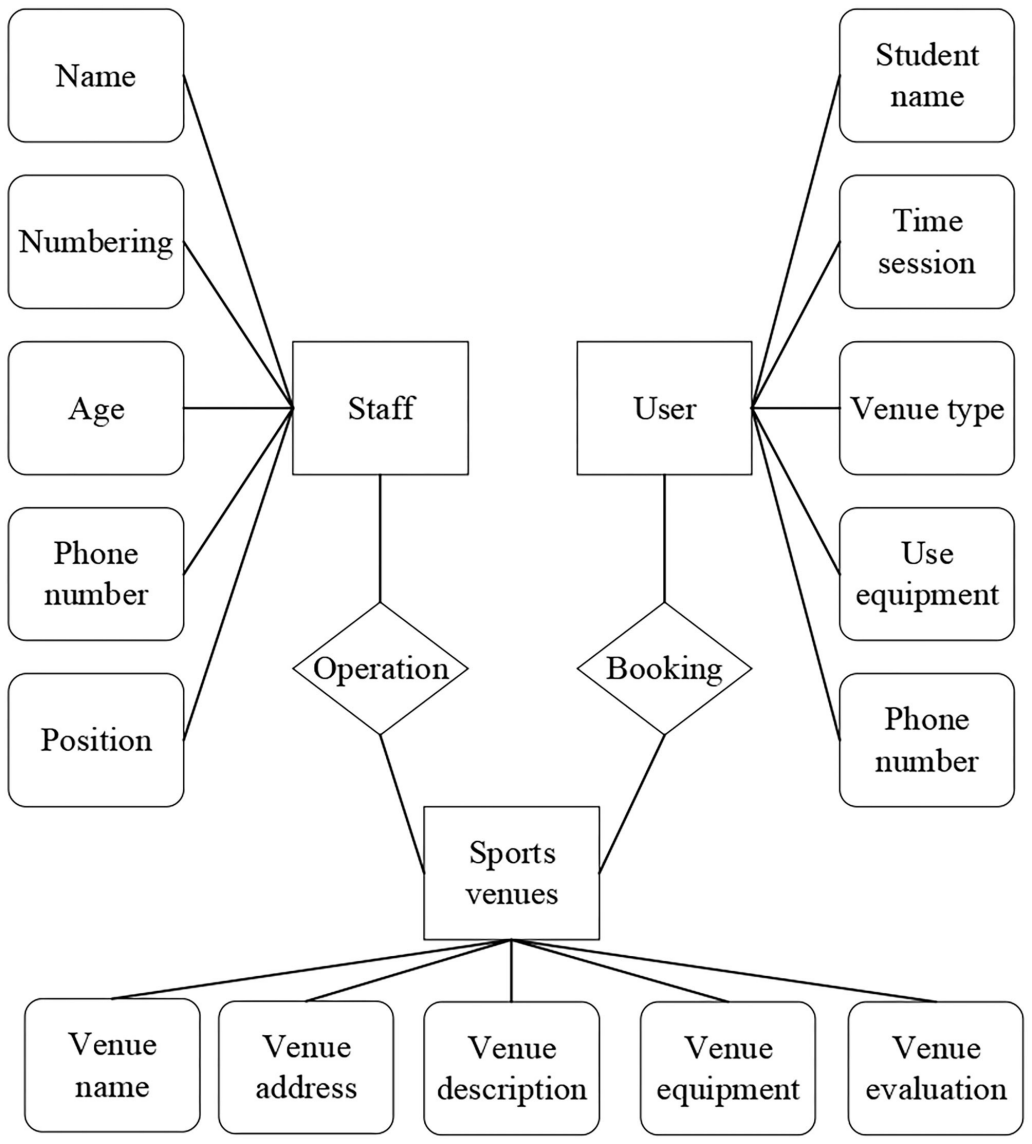
E-R diagram of venue information.

An entity relationship (E-R) diagram is composed of entity, attribute, and relation. The following Figure 4 is the E-R diagram of the two main modules of venue information and course information.

**Figure 5:**
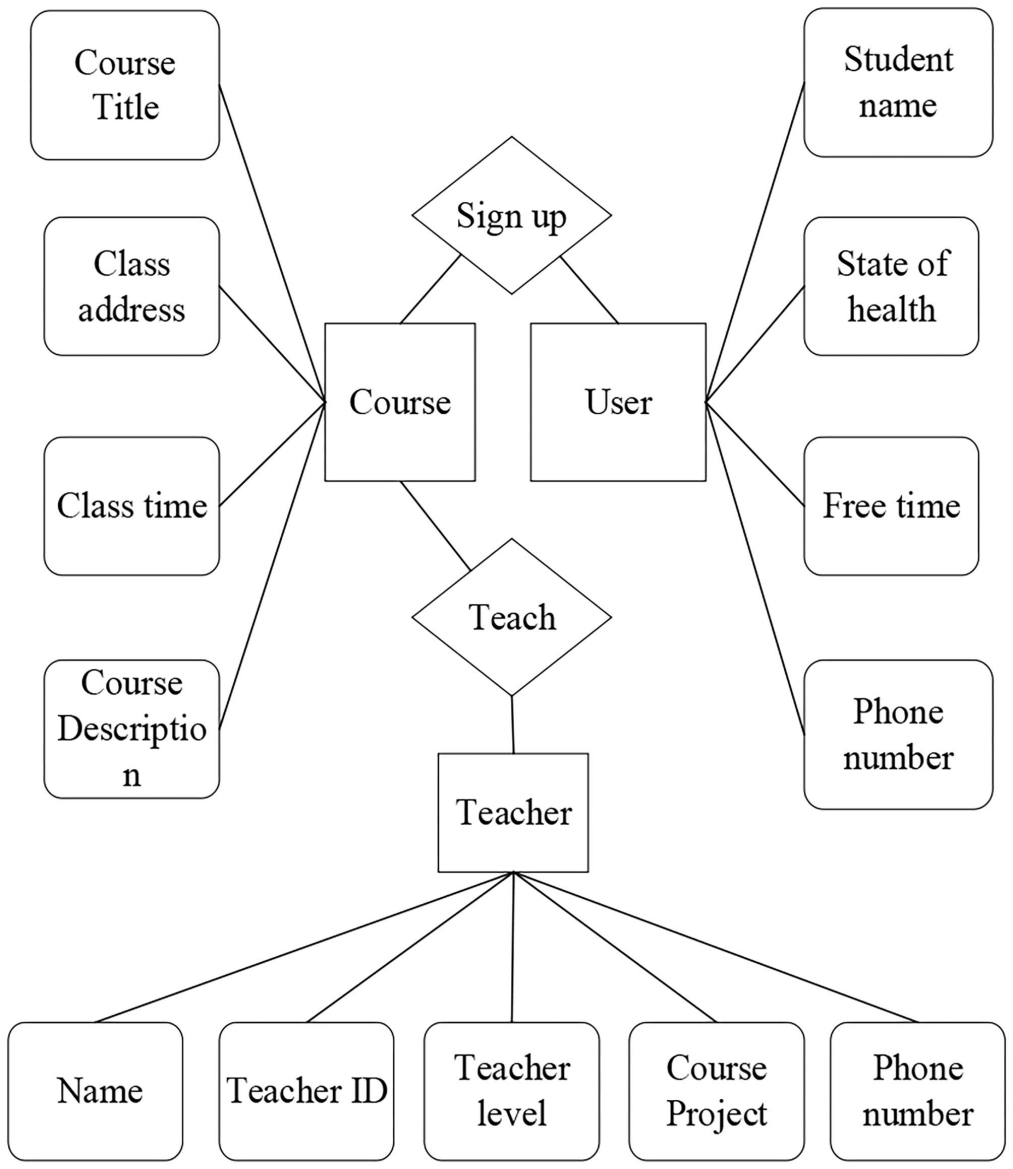
E-R diagram of course information.

Each entity of the venue module and its ownership attributes are explained as follows in Figure 5.

(1) User-included attributes: student name, phone number, venue type, time session, venue, and equipment used;
(2) Venue-included attributes: venue name, venue address, venue description, venue service, and venue evaluation;
(3) Staff-included attributes: number, name, age, position, and phone number.

Each entity of the course module and its attributes are explained as follows.

(1) User-included attributes: student name, phone number, state of health, and free time;
(2) Course-included attributes: course name, course address, course description, and course price;
(3) Teacher-contained attributes: teacher ID, name, phone number, teacher level, and teaching program.

Intelligent sports management system architecture is a closed-loop structure, including the user (student) end, venue end, athlete end, and PC management system. In the student end, the school can easily and quickly carry out some operations and enjoy the learning stadium services through the system, mainly made up of the home page, venues, courses, sports, and personal home page. The home page mainly includes city selection and weather and temperature information display, sports scheme recommendation and view, fast retrieval of required venues, sports list and news announcement, notice and private messages, and other functional modules. The venue module mainly includes the functional modules of venue reservation, venue retrieval, and venue recommendation. The course module mainly includes course retrieval, course recommendation, course reservation, and other functional modules. The sports module includes the development and recommendation of sports programs, the view of the sports program, the exchange and sharing of some information, news, and sports experience about the sports circle, and other functional modules. The personal home page module mainly includes account management, data setting and modification, my account, venue reservation and course registration information, and recommendation and sharing modules.

### The Relationship Between Sports Lifestyle and Mental Health of College Students

Sports lifestyle refers to the stable situation and behavior characteristics of all sports activities, which are caused by certain values of individuals, groups, or all members of the society, and meet these multi-level needs under certain social conditions. The sports lifestyle of college students is closely related to the sports training environment instructional system in colleges and universities. In exploring the influencing factors of mental health, sports lifestyle has become an important variable to explain the differences in health amongst students. It has become a common understanding to use sports lifestyle to promote and change people's mental health. Kim provided a physiological basis for the relationship between sports lifestyle and mental health, and studied that sports lifestyle could improve their neurological function, maintain a good state of mind, and relieve tension, anxiety, and depression (Kim et al., 2020). Throughout the research process of sports lifestyle and mental health, previous research on sports lifestyle mostly used self-made questionnaires, which only made statistical analysis on sports time, frequency, intensity, and sports events; the summary of sports lifestyle was incomplete, and there was almost no research on the relationship between sports lifestyle and mental health. Based on this situation, the authoritative physical activity rating scale and the internationally recognized symptom checklist 90 (SCL-90) questionnaire are used to study the relationship between sports lifestyle and the mental health of contemporary college students. On the one hand, it can make college students pay attention to their own sports lifestyle and their own mental health; on the other hand, the influence of sports lifestyle on mental health can be studied, which can indirectly promote mental health by guiding students' sports life, and provide new ideas for the promotion of college students' mental health.

### Research on the Effect of Intelligent Sports System on College Students' Mental Health

Shanghai X University is selected as the source of the respondents. The Physical Activity Rating Scale-3 (PARS-3), Self-Rating Depression Scale (SDS), and Self-Rating Anxiety Scale (SAS) are used. The class is taken as a whole, and random cluster sampling is conducted to analyze the differences of depression and anxiety among college students with different amounts of exercise. A total of 569 electronic questionnaires are distributed and 510 are recovered. After the invalid questionnaires, such as those with random filling, are eliminated, 496 valid questionnaires are obtained.

### Questionnaire Design and Survey Tools

PARS-3 scale is compiled by Japanese psychologist Takao Hashimoto. The Chinese version of the scale is used in the survey, which mainly reflects the physical exercise in the past month. Domestic research shows that the Chinese version of PARS scale can better evaluate the amount of physical exercise of ordinary college students; its internal consistency reliability is 0.796~0.856, split half reliability is 0.794~0.8, and test-retest reliability is 0.82. The scale involves three indexes: intensity, time, and frequency of physical exercise. Each index is scored by five levels. The intensity and frequency are respectively “1–5 points,” and the time is “0–4 points.” The higher the intensity is, the longer the time is and the higher the frequency is, the higher the score is and the greater the amount of exercise is. The calculation equation of the amount of exercise is “intensity × time × frequency,” so the score range of the scale is “0~100 points.” According to the relevant research of domestic predecessors, the classification standard of college students' exercise amount is as follows: “ ≤ 19 points is a small amount of exercise (insufficient physical exercise), 20–42 points is a medium amount of exercise, and ≥ 43 points is a large amount of exercise.” SCL-90 is used as the mental health scale. The validity of each symptom is 0.77–0.99, which has good reliability and validity. The scale is divided into 10 dimensions of somatization, obsessive-compulsive symptoms, interpersonal sensitivity, depression, anxiety, hostility, phobia, paranoia, psychoticism, and other, with a total of 20 items. For each question, five levels of scoring standards are set up from “no” to “serious,” respectively, which are assigned as “1,” “2,” “3,” “4,” and “5” points. The lowest total score is 90 points, and the highest score is 450 points. The lower the score is, the lighter the symptoms are, and the better the psychological condition is.

## Results and Discussion

### Application of Artificial Intelligence Sports Scheme Recommendation Algorithm

**Figure 6:**
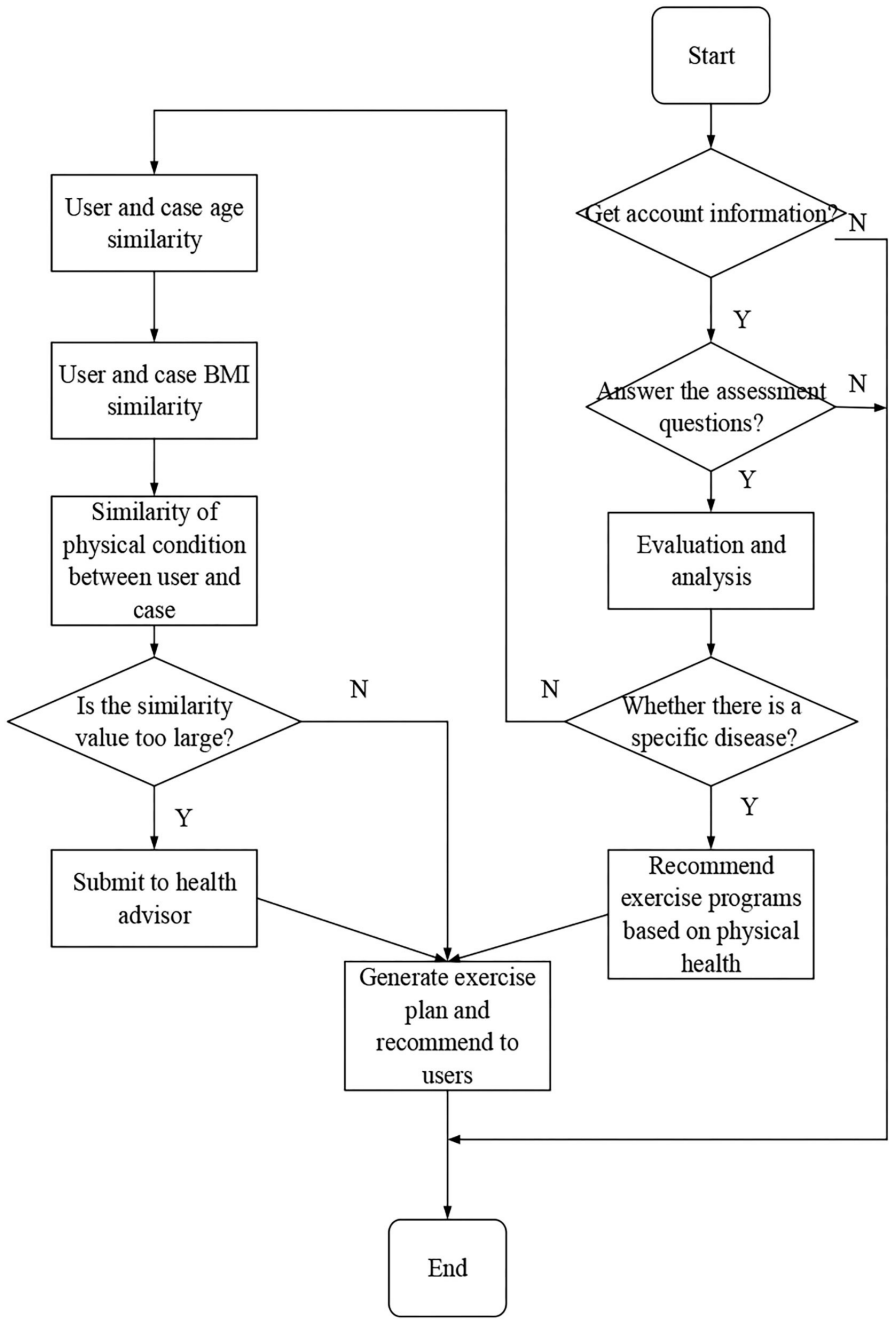
Flow system of sports scheme making and recommendation module.

When the users want to obtain the sports scheme, the intelligent sports management system will prompt them to answer several simple questions to make an evaluation, and the system will obtain some personal information of their registered account to use. Once the users are not allowed to access, the following series of operations are not carried out, and users' choice is respected. APP will not take the initiative to use some users' personal information and push sports programs to them. Only when users know and allow it will the system will obtain some information of their account, such as age, height, weight, and gender, and calculate and analyze it. If users do not fill in the relevant information when registering the account, the system will add some corresponding questions in the evaluation stage to let them answer. After the analysis and evaluation of the information, the system will make a score and classification for users, mainly including the score of chest muscle, heart and lung, abdomen, flexibility, and lower limbs. By scoring, the system can know some conditions of users, such as sports ability and whether they usually exercise or not. According to the classification of users, combined with the completed knowledge base, using the reasoning method based on case and rule, the system selects the sports scheme and recommends it to users. Figure 6 is the flow system of sports scheme making and recommendation module.

### Implementation of Course Registration Module of Intelligent Sports System

**Figure 7:**
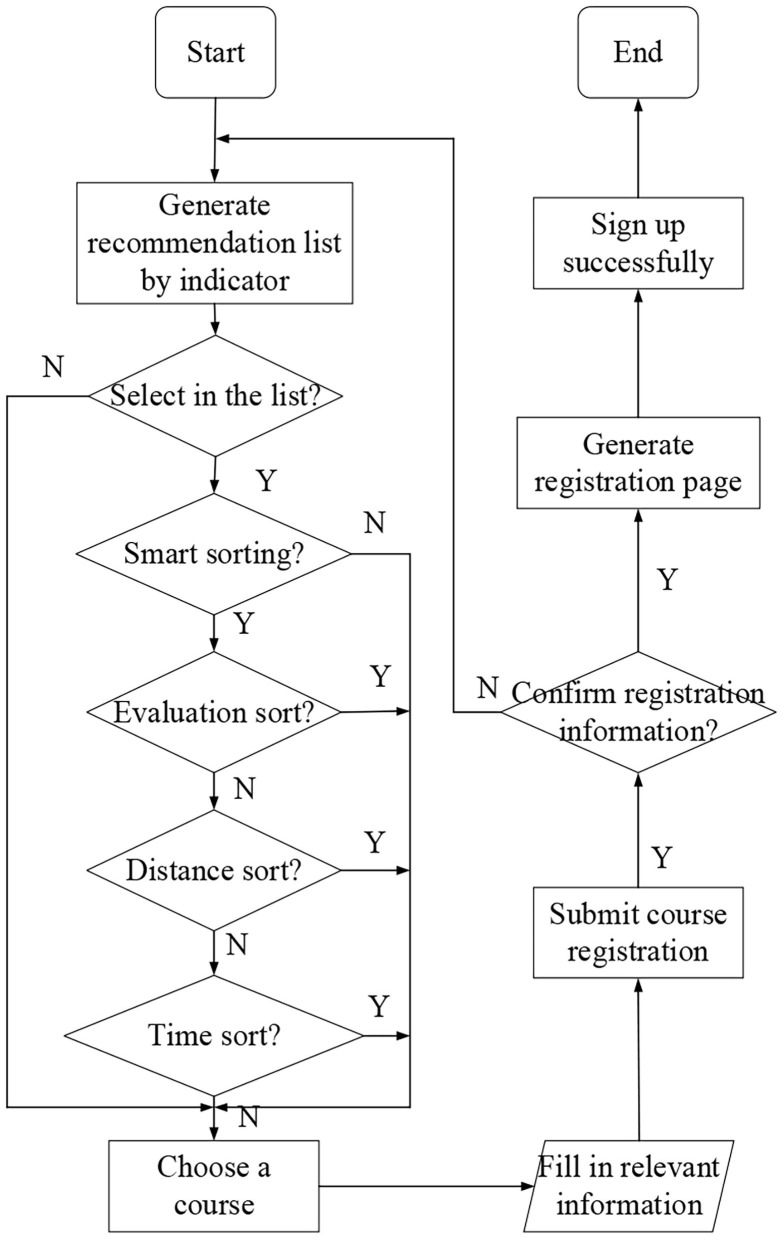
Flow chart of course registration module.

The course recommendation and course registration module is mainly for the registered users. Before enrolling for the course, users should first find courses that meet their own requirements. The system can generate the course recommendation list through the recommendation model according to the user's basic information and course information. Users view courses according to the course recommendation list. At the same time, they can also display the course list by manually selecting the course type and sorting the list conditions. The default sorting method is intelligent sorting, which is mainly used to display the courses that meet the user's requirements according to the user's information, and then according to distance, opening time, course evaluation, and so on. After obtaining the course list, users can select the courses that meet their requirements through the detailed information of courses. The detailed information of the course includes course time, teaching address, course introduction, course evaluation, curriculum schedule, and course requirements, which help users better understand the course. After finding the right course, users can sign up for the course and wait for the class opening notice. The course opening notice will be informed to users by a short message service and client message. Figure 7 shows the flow chart of course recommendation and course registration.

### Database Model Design of Artificial Intelligence Sports Management System

**Figure 8:**
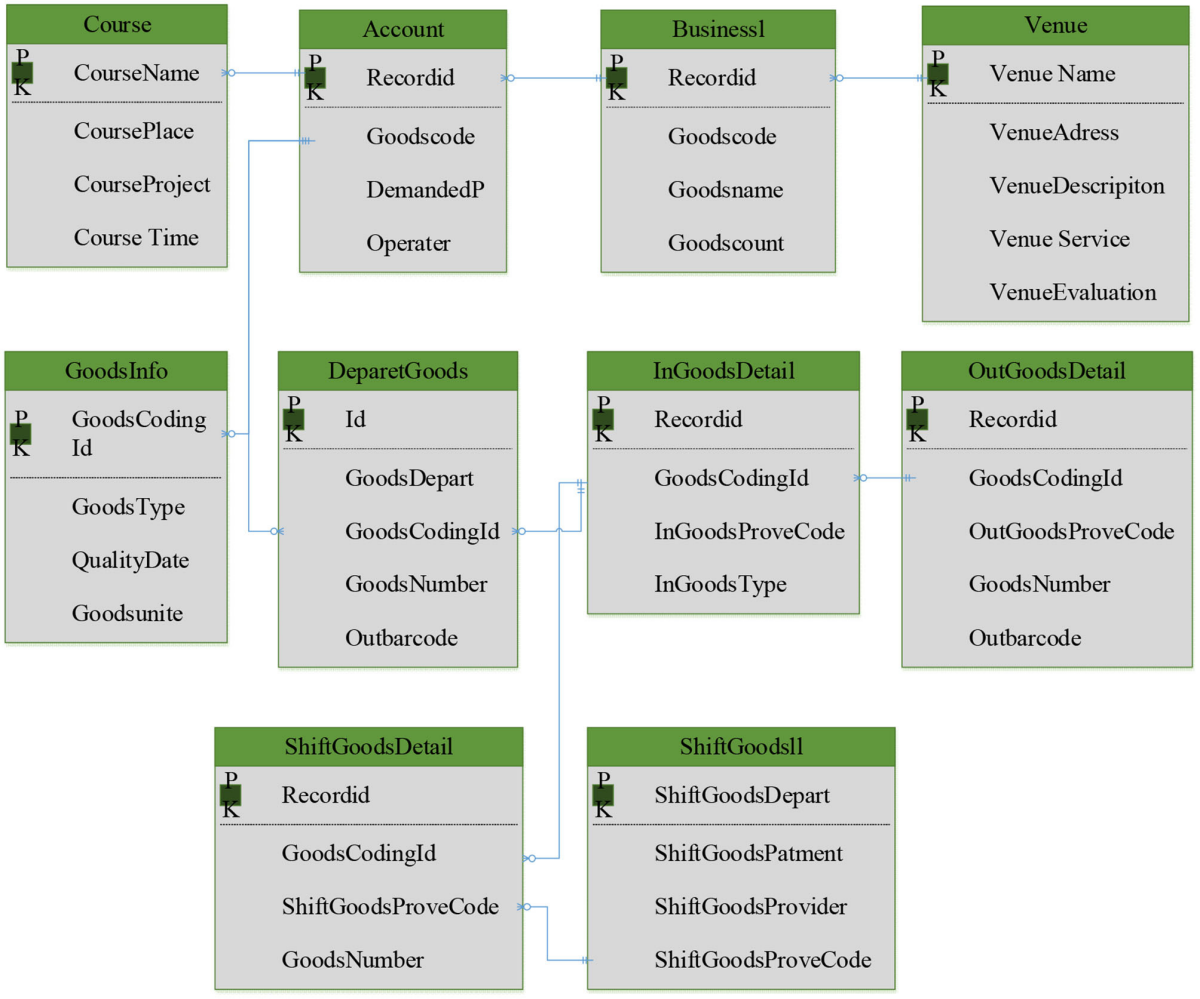
Relationship model of system database.

On the basis of fully understanding the process of an intelligent sports management system, the system data flow is analyzed, and then the corresponding database relational model is sorted out. After the understanding of entities, relationships among entities, and their attributes, the data types and storage methods of each entity are analyzed. E-R diagram is used to design a database relational model. Figure 8 shows the system relationship model.

### System Test

After the development of the system, it is used to test whether the function of the system is consistent with the function specification written in the requirement analysis. Function test case list usually includes use case identification, test project, test method, test item, input/ operation, expected result, actual result, and so on. The purpose of use case identification is to distinguish between positioning and modification at that time. Input/operation refers to the introduction and description of functions, and then the input and operation steps are briefly described. Finally, the actual test results are compared with the expected results to see if there is any difference. The following is the intelligent sports management system venue booking use cases, as shown in Table 1 below.

**Table 1:** Booking system functional test.

**Use case identification**	**OnlingBooking_001**
Test project	Venue reservation
Test method	BLACK BOX
Test item	Basic operation and data verification of venue reservation
Input/operation	Open the user app; Search for the required venues; Identify venue types and projects; Check the booking status; Choose sports time; Specific site number; Submit booking information.
Expected result	Display the correct venue information; Show the daily booking situation; Normal selection of idle sessions; Display correct booking information after submission.
Actual results	Booking submitted successfully; Consistent with the actual results; Consistent with the PC display.

### Effect of Intelligent Sports System on Mental Health of College Students

The data analysis of the questionnaire shows that 64% of sample students increased their weekly exercise frequency after the promotion of the school intelligent sports training system. Table 2 shows the differences in anxiety and depression scores of college students with different amounts of exercise.

**Table 2:** The influence of different amount of exercise on college students' anxiety.

**Amount of exercise**		**Anxiety score**
Small amount of exercise		45.32
Medium amount of exercise		43.58
Large amount of exercise		38.49
Group comparison		*p* < 0.001^***^
Multiple comparisons (LSD correction)	Small and medium	*p* < 0.001^***^
	Small and large	*p* < 0.001^***^
	Medium and large	*p* = 0.154

*** *p < 0.001*.

As shown in Table 2, there are significant differences in anxiety scores among groups with different amounts of exercise (*F* = 3.64, *p* < 0.001). Compared with the group with a small amount of exercise, the anxiety scores of groups with medium and large amounts of exercise are significantly lower (LSD correction, *p* < 0.001).

As shown in Table 3, there are significant differences in depression scores among groups with different amounts of exercise (*F* = 26.42, *p* < 0.001). Compared with the group with a small amount of exercise, the groups with medium and large amounts of exercise have significantly lower depression scores (LSD correction, *p* < 0.001). Compared with the group with a medium amount of exercise, the depression score of the group with a large amount of exercise is significantly lower (LSD correction, *p* = 0.003).

**Table 3:** Effect of different exercise amount on depression of college students.

**Amount of exercise**		**Depression score**
Small amount of exercise		52.94
Medium amount of exercise		47.68
Large amount of exercise		41.36
Group comparison		*p* < 0.001^***^
Multiple comparisons (LSD correction)	Small and medium	*p* < 0.001^***^
	Small and large	*p* < 0.001^***^
	Medium and large	*p* = 0.005^**^

** *p < 0.05*;

*** *p < 0.001*.

## Conclusion

Based on the B/S structure, the intelligent sports management system is constructed. The basic framework of Spring Cloud is used to integrate the framework and components of each part, and a distributed microservice system is built; age, BMI index, and physical health status are analyzed, the same sports scheme is recommended to users, and the intelligent sports program recommendation function is realized. After the entity and the relationship between the entities of the intelligent sports system are analyzed, the database relational model of the system is designed with E-R diagram. The functional test results show that the system can accurately display the venue information, complete the online booking, and show the compatible effect on multiple systems. The data analysis results of the questionnaire survey show that there are significant differences in the scores of anxiety and depression among groups with different amounts of exercise; the larger the amount of exercise is, the lower the scores of anxiety and depression are.

The design goal of this system is to use artificial intelligence information technology to develop a set of college sports venues' network intelligent management systems. By installing a browser on the mobile phone or computer, users can log in to the college venue system; college students can achieve registration. Physical education teachers in colleges and universities can release course information and provide guidance for college students' extracurricular exercise, so that there is no conflict between extracurricular exercise and physical education in the venues, and the utilization rate of venue resources can be improved. College students can better carry out sports in class and after class, which can improve their physical fitness and stimulate their sports vitality and physical and mental health.

In this exploration, the main functions of the designed intelligent sports training management system in colleges and universities can be basically realized, but there are still many problems to be improved. It is necessary to optimize the system continuously from the aspects of function, performance, operability, compatibility, and security; with the accumulation of system data, it is necessary to consider the use of big data, data mining, and other advanced technologies to establish a data warehouse to conduct statistical analyses of various data of college students' sports training, so as to provide data support for school sports management decision-making.

## Data Availability Statement

The datasets presented in this article are not readily available because The dataset was restricted by the university labs regulations. Requests to access the datasets should be directed to Ting Wang, ktwphd83@sina.com.

## Ethics Statement

The studies involving human participants were reviewed and approved by Catholic Kwandong University Committee. The patients/participants provided their written informed consent to participate in this study. Written informed consent was obtained from the individual(s) for the publication of any potentially identifiable images or data included in this article.

## Author Contributions

All authors listed have made a substantial, direct and intellectual contribution to the work, and approved it for publication.

## Conflict of Interest

The authors declare that the research was conducted in the absence of any commercial or financial relationships that could be construed as a potential conflict of interest.

## References

[B1] Agans J. P. Wilson O. W. A. Bopp M. (2020). Required health and wellness courses: associations with college student physical activity behavior and attitudes. J. Phys. Act. Health 17, 632–640. 10.1123/jpah.2019-0362 32369760

[B2] Bharathi R. Selvarani R. (2019). A machine learning approach for quantifying the design error propagation in safety critical software system. IETE J. Res. 1–15. 10.1080/03772063.2019.1611490

[B3] Chen M. (2019). The impact of expatriates' cross-cultural adjustment on work stress and job involvement in the high-tech industry. Front. Psychol. 10:2228. 10.3389/fpsyg.2019.02228 31649581 PMC6794360

[B4] Gómez O. S. Rosero R. H. Cortés-Verdín K. (2020). CRUDyLeaf: a DSL for generating spring boot REST APIs from entity CRUD operations. IICT BAS 20, 3–14. 10.1007/978-1-4842-2931-6_11

[B5] Kim H. N. Kwon Y. B. Jun E. J. Kim J. B. (2020). Health-risk behavior-, mental health-, and physical exercise-related risk factors for tooth fractures in Korean adolescents. Int. J. Environ. Health Res. 17:7815. 10.3390/ijerph17217815 33114513 PMC7662374

[B6] Kravari K. Bassiliades N. (2019). StoRM: a social agent-based trust model for the internet of things adopting microservice architecture. Simul. Model. Pract. Theory 94, 286–302. 10.1016/j.simpat.2019.03.008

[B7] Leffler A. J. Beard K. H. Kelsey K. C. Choi R. T. Schmutz J. A. Welker J. M. (2019). Cloud cover and delayed herbivory relative to timing of spring onset interact to dampen climate change impacts on net ecosystem exchange in a coastal Alaskan wetland. Environ. Res. Lett. 14:084030. 10.1088/1748-9326/ab1c91

[B8] Li J. Wang W. C. Mao J Wang Z. Zeng G. Chen G. (2019). Persistent spring shortwave cloud radiative effect and the associated circulations over southeastern China. J. Clim. 32, 3069–3087. 10.1175/JCLI-D-18-0385.1

[B9] Li M. Ning C. (2020). The reform of modern university sports teaching mode based on the evaluation standard. Solid State Technol. 63, 481–489. 10.2991/iccese-19.2019.208

[B10] Li Y. Qu C. (2019). College english education platform based on browser/server structure and flipped classroom. Int. J. Emerg. Technol. Learn. 14, 171–181. 10.3991/ijet.v14i15.11147

[B11] Lin F. Zhou Y. You I. Lin J. An X. Lü X. (2019). Content recommendation algorithm for intelligent navigator in fog computing based IoT environment. IEEE Access 7, 53677–53686. 10.1109/ACCESS.2019.2912897

[B12] Morrison A. L. Kay J. E. Frey W. R. Chepfer H. Guzman R. (2019). Cloud response to Arctic Sea ice loss and implications for future feedback in the CESM1 climate mode. J. Geophys. Res. Atmos. 124, 1003–1020. 10.1029/2018JD029142

[B13] Qian J. Song B. Jin Z. Wang B. Chen H. (2018). Linking empowering leadership to task performance, taking charge, and voice: the mediating role of feedback-seeking. Front. Psychol. 9:2025. 10.3389/fpsyg.2018.02025 30410461 PMC6209672

[B14] Renó V. Dimauro G. Labate G. Stella E. Fanizza C. Cipriano G.. (2019). A SIFT-based software system for the photo-identification of the Risso's dolphin. Ecol. Inform. 50, 95–101. 10.1121/1.4785105

[B15] Tian Y. Zheng B. Wang Y. Zhang Y. Wu Q. (2019). College library personalized recommendation system based on hybrid recommendation algorithm. Procedia CIRP 83, 490–494. 10.1016/j.procir.2019.04.126

[B16] Vaughn M. Hur J. W. Russell J. (2019). Flipping a college physical activity course: impact on knowledge, skills, and physical activity. Int. J. Educ. Res. 3, 87–98. 10.33902/jpr.vi0.126

[B17] Wu W. Wang H. Wu Y. (2020). Internal and External Networks, and Incubatees' Performance in Dynamic Environments: Entrepreneurial Learning's Mediating Effect. J. Technol. Transf. 10.1007/s10961-020-09790-w

[B18] Wu W. Wang H. Zheng C. Wu Y. J. (2019). Effect of narcissism, psychopathy, and machiavellianism on entrepreneurial intention—the mediating of entrepreneurial self-efficacy. Front. Psychol. 10:360. 10.3389/fpsyg.2019.00360 30846958 PMC6393355

[B19] Wu Y. Song D. (2019). Gratifications for social media use in entrepreneurship courses: learners' perspective. Front. Psychol. 10:1270. 10.3389/fpsyg.2019.01270 31214081 PMC6555126

[B20] Wu Y. Wu T. (2017). A decade of entrepreneurship education in the Asia Pacific for future directions in theory and practice. Manag. Decis. 55, 1333–1350. 10.1108/MD-05-2017-0518

[B21] Yan L. Cao S. Gong Y. Han H. Wei J Zhao Y.. (2019). SatEC: a 5G satellite edge computing framework based on microservice architecture. Sensors 19:831. 10.3390/s19040831 30781604 PMC6412722

[B22] Yang Y. Zhao C. Fan H. (2020). Spatiotemporal distributions of cloud properties over China based on Himawari-8 advanced Himawari imager data. Atmos. Res. 240:104927. 10.1016/j.atmosres.2020.104927

[B23] Yi Z. Meilin W. RenYuan C. YangShuai W. Jiao W. (2019). Research on application of SME manufacturing cloud platform based on micro service architecture. Procedia CIRP 83, 596–600. 10.1016/j.procir.2019.04.091

[B24] Yin C. Shi L. Sun R. Wang J. (2020). Improved collaborative filtering recommendation algorithm based on differential privacy protection. J. Supercomput. 76, 5161–5174. 10.1007/s11227-019-02751-7

[B25] Zhao C. Chen Y. Li J. Letu H. Su Y. Chen T.. (2019). Fifteen–year statistical analysis of cloud characteristics over China using Terra and Aqua Moderate Resolution Imaging Spectroradiometer observations. Int. J. Climatol. 39, 2612–2629. 10.1002/joc.5975

[B26] Zhao Z. Yang J. (2019). Design and implementation of computer aided physical education platform based on Browser/Server architecture. Int. J. Emerg. Technol. Learn. 14, 40–51. 10.3991/ijet.v14i15.11146

[B27] Zheng W. Wu Y. Chen L. (2018). Business intelligence for patient-centeredness: a systematic review. Telemat. Inform. 35, 665–676. 10.1016/j.tele.2017.06.015

