# Supplementary material for: Design and Implementation of Intelligent Sports Training System for College Students' Mental Health Education
Source: Front Psychol. 2021 Apr 8;12:634978. doi: 10.3389/fpsyg.2021.634978 (PMC8060568; doi:10.3389/fpsyg.2021.634978)
Supplement: Supplementary file 2 [file Data_Sheet_2.docx]

Dear everyone,

Hello! Welcome to participate in the questionnaire on “the use of intelligent sports training system and college students' physical exercise methods and mental health”. The purpose of this questionnaire is to investigate the learning of the use of intelligent sports teaching environment system and the current situation of college students' physical exercise methods and mental health. The private part of the questionnaire will be kept completely confidential. There is no right or wrong question in this questionnaire. Please answer all the questions according to your real ideas and the questionnaire requirements.

Thank you for your support!

**Part 1: basic information investigation**

1. Gender: A. male B. female

2. Grade: A. freshman B. sophomore C. junior D. senior

3. Age: ____

4. Your evaluation of your health: A. very poor B. general C. relatively good D. good

5. Club you belong to: A. none B. sports club C. other clubs

**Part 2: intelligent sports training system and physical exercise**

6. Sports you often take part in (please choose 1-3 items):

A. basketball B. football C. badminton D. tennis E. table tennis F. volleyball G. walking H. jogging I. track and field J. swimming K. roller skating L. aerobics M. dance N. martial arts O. others

7. Frequency of weekly exercise: A. 0-once B. twice C. three times D. four times E. five times and above

8. The average number of times you book venues and participate in sports through the school's intelligent sports training system per week:

A. 0-once B. twice C. three times D. four times E. five times and above

9. Your average exercise time: A. less than 15 minutes B. 15-30 minutes C. 30-45 minutes D. 45-60 minutes E. more than 60 minutes

10. Your average exercise intensity each time: A. no sweat B. slight sweat C. moderate sweat D. sweat more E. sweat profusely

11. After the promotion of school intelligent sports training system, how about your weekly exercise frequency?

A. increases B. decreases C. unchanged

**Part 3: mental health status**

Guidelines: problems that some people may encounter are listed as follows. Please read each item carefully, and then mark the score of each question according to the actual feeling affected by the following situation in the last week. Among them, "no" is 1, "very light" is 2, "medium" is 3, "heavy" is 4, "serious" is 5. Please fill in according to your own situation. Thank you!

| **Questions** | **Choices** |
| --- | --- |
| 1. I feel unhappy and depressed. | 1-2-3-4-5 |
| 2. I burst out crying sometimes or feel like crying. | 1-2-3-4-5 |
| 3. There are unnecessary thoughts or words in the brain. | 1-2-3-4-5 |
| 4. I feel tired for no reason. | 1-2-3-4-5 |
| 5. I don't find it difficult to do what I often do. | 1-2-3-4-5 |
| 6. I do not sleep well at night. | 1-2-3-4-5 |
| 7. I have hope for the future. | 1-2-3-4-5 |
| 8. My life is very interesting. | 1-2-3-4-5 |
| 9. I feel like a useful person, and someone needs me. | 1-2-3-4-5 |
| 10. I think if I died, other people would live a better life. | 1-2-3-4-5 |
| 11. I feel more nervous and anxious than usual. | 1-2-3-4-5 |
| 12. I am scared for no reason. | 1-2-3-4-5 |
| 13. I am easily upset or frightened. | 1-2-3-4-5 |
| 14. I feel weak and tired easily. | 1-2-3-4-5 |
| 15. I suffer from headache, head and neck pain and backache. | 1-2-3-4-5 |
| 16. I think everything is fine and nothing bad will happen. | 1-2-3-4-5 |
| 17. My hands and feet are shaking. | 1-2-3-4-5 |
| 18. I feel that most people cannot be trusted. | 1-2-3-4-5 |
| 19. I blush and get hot. | 1-2-3-4-5 |
| 20. I feel calm and easy to sit quietly. | 1-2-3-4-5 |
